# *STM2209-STM2208 (opvAB)*: A Phase Variation Locus of *Salmonella enterica* Involved in Control of O-Antigen Chain Length

**DOI:** 10.1371/journal.pone.0036863

**Published:** 2012-05-11

**Authors:** Ignacio Cota, Anne Béatrice Blanc-Potard, Josep Casadesús

**Affiliations:** 1 Departamento de Genética, Facultad de Biología, Universidad de Sevilla, Sevilla, Spain; 2 Unité Mixte de Recherches 5235, Centre National de la Recherche Scientifique et Université Montpellier II, Montpellier, France; Indian Institute of Science, India

## Abstract

*STM2209* and *STM2208* are contiguous loci annotated as putative protein-coding genes in the chromosome of *Salmonella enterica*. Lack of homologs in related Enterobacteria and low G+C content suggest that *S. enterica* may have acquired *STM2209-STM2208* by horizontal transfer. *STM2209* and *STM2208* are co-transcribed from a promoter upstream *STM2209*, and their products are inner (cytoplasmic) membrane proteins. Analysis with the bacterial adenylate cyclase two-hybrid system suggests that STM2209 and STM2208 may interact. Expression of *STM2209-STM2208* is subjected to phase variation in wild type *Salmonella enterica* serovar Typhimurium. Switching frequencies in LB medium are 6.1×10^−5^ (OFF→ON) and 3.7×10^−2^ (ON→OFF) per cell and generation. Lack of DNA adenine methylation locks *STM2209-STM2208* in the ON state, and lack of the LysR-type factor OxyR locks *STM2209-STM2208* in the OFF state. OxyR-dependent activation of *STM2209-STM2208* expression is independent of the oxidation state of OxyR. *Salmonella* cultures locked in the ON state show alteration of O-antigen length in the lipopolysaccharide, reduced absorption of bacteriophage P22, impaired resistance to serum, and reduced proliferation in macrophages. Phenotypic heterogeneity generated by *STM2209-STM2208* phase variation may thus provide defense against phages. In turn, formation of a subpopulation unable to proliferate in macrophages may restrain *Salmonella* spread in animal organs, potentially contributing to successful infection.

## Introduction

Phase variation, the reversible switch of gene expression at high frequency (e. g., >10^−5^ per cell and generation), is a common phenomenon in bacteria (reviewed in [1], [2]). Switching turns gene expression from OFF to ON, or from low expression to high expression, and *vice versa*. A consequence of phase variation is phenotypic heterogeneity in clonal bacterial populations, a phenomenon of paramount relevance for bacterial survival in harsh environments. In bacterial pathogens, for instance, phenotypic heterogeneity in cell envelope components may facilitate immune evasion and modulation [2], [3]. Classical examples of phase variation in pathogenic bacteria involve loci encoding surface-exposed proteins, cell appendixes such as fimbriae, pili, and flagella, and lipopolysaccharide modification functions [1], [2], [4], [5]. Phase variation, however, is not restricted to bacterial pathogens nor to loci that encode components of the cell surface [6], [7].

Bacteria use a variety of mechanisms to produce phase variation [1], and a relatively common type of control involves switching between alternative epigenetic states. Each epigenetic state is propagated by a feedback loop, and reversed after a certain number of generations. Epigenetic regulation of phase variation systems is often controlled by DNA adenine methylation (reviewed in [8], [9]). Paradigms of this kind of regulation are the *pap* operon of uropathogenic *E. coli*, which encodes fimbriae for attachment to the urinary epithelium [10], and the *agn43* gene of *E. coli*, which encodes a non-fimbrial adhesin [11]. Other phase variation loci under Dam methylation control are the glycosyltransferase operon (*gtr*) of phage P22 [4], the *clp* operon of enterotoxigenic *E. coli* [12], the *pef* operon of the *Salmonella* virulence plasmid [13], and perhaps the *S. enterica std* fimbrial operon [14].

This study describes a new phase variation locus in *Salmonella enterica* serovar Typhimurium. The locus, annotated as *STM2209-STM2208* in the *Salmonella* genome database [15], is present in *Salmonella enterica* but not in *Salmonella bongori* nor in other enteric bacteria. Aside from the annotation of *STM2209-STM2208* as putative protein-coding genes, the literature contains little information on *STM2209-STM2208*. An exception is a transcriptome analysis in Dam^+^ and Dam^−^ strains of *S. enterica* which revealed that *STM2209-STM2208* transcripts are more abundant in a Dam^−^ background [16]. This observation tentatively classified *STM2209-STM2208* as a locus repressed by Dam methylation [16]. However, we show that *STM2209-STM2208* is actually a phase variation locus whose expression is locked in the ON state in Dam^−^ mutants. We also show that lack of the LysR-like factor OxyR locks *STM2209-STM2208* expression in the OFF state. *STM2209* and *STM2208* are part of a single transcriptional unit, and encode inner membrane proteins. Constitutive expression of *STM2209-STM2208* alters lipopolysaccharide O chain length, reduces phage P22 adsorption, decreases resistance to serum, and impairs proliferation in macrophages. Altogether, our observations suggest that phase variation of *STM2209-STM2208* may contribute to phenotypic heterogeneity in *Salmonella* populations, providing defense against phages and restraining *Salmonella* spread in animal organs.

## Methods

### Bacterial strains, plasmids, bacteriophages, and strain construction

All the strains of *Salmonella enterica* used in this study (Table 1) belong to serovar Typhimurium, and derive from ATCC 14028. For simplicity, *S. enterica* serovar Typhimurium is often abbreviated as *S. enterica*. *Escherichia coli* BTH101 (F^−^ *cya-99 araD139 galE15 galK16 rpsL1*(Str^r^) *hsdR2 mcrA1 mcrB1*) was used for bacterial two-hybrid assays. *E*. *coli* CC118 lambda *pir* [*phoA20 thi-1 rspE rpoB argE(Am) recA1 (*lambda *pir)*] and *E. coli* S17-1 lambda *pir* [*recA pro hsdR* RP4-2-Tc::Mu-Km::Tn*7 (*lambda *pir)*] were used for directed construction of point mutations. Plasmids constructed for this study are listed in Table 2.

**Table 1:** Strains of *Salmonella enterica* serovar Typhimurium.

Strain	Genotype
ATCC 14028	wild type
SV4536	Δ*dam*-230
SV5573	*STM2208*::3xFLAG
SV5574	Δ*dam*-230 *STM2208*::3xFLAG
SV5676	Δ*STM2209*::*lac* (transcriptional)
SV5677	Δ*STM2208*::*lac* (transcriptional)
SV5679	Δ*STM2208*::*lac* (translational)
SV5680	Δ*dam*-230 Δ*STM2209*::*lac* (transcriptional)
SV5681	Δ*dam*-230 Δ*STM2208*::*lac* (transcriptional)
SV5683	Δ*dam*-230 Δ*STM2208*::*lac* (translational)
SV5734	Δ*STM2209*::*lac* (translational)
SV5735	Δ*dam*-230 Δ*STM2209*::*lac* (translational)
SV5812	*STM2209*::3xFLAG
SV5813	Δ*dam*-230 *STM2209*::3xFLAG
SV5925	Δ*oxyR*::Cm^r^
SV5989	Δ*oxyR*::Cm^r^ Δ*STM2208*::*lac* (translational)
SV5990	Δ*dam*-230 Δ*oxyR*::Cm^r^ Δ*STM2208*::*lac* (translational)
SV6001	Δ*oxyR*::Cm^r^ *STM2208*::3xFLAG
SV6002	Δ*dam*-230 Δ*oxyR*::Cm^r^ *STM2208*::3xFLAG
SV6004	Δ*oxyR*::Cm^r^ *STM2209*::3xFLAG
SV6005	Δ*dam*-230 Δ*oxyR*::Cm^r^ *STM2209*::3xFLAG
SV6013	Δ*STM2209*-*STM2208*
SV6397	*oxyR* ^C199S^
SV6401	mut. GATC
SV6976	mut. GATC Δ*STM2209*-*STM2208*
SV7031	mut. GATC Δ*STM2208*::*lac* (translational)
SV7032	Δ*dam*-230 mut. GATC Δ*STM2208*::*lac* (translational)
SV7232	Δ*oxyR*::Cm^r^ mut. GATC Δ*STM2208*::*lac* (translational)
SV7233	Δ*dam*-230 Δ*oxyR*::Cm^r^ mut. GATC Δ*STM2208*::*lac* (translational)

**Table 2:** Plasmids constructed for this study.

Plasmid number	Description
pIZ1758	pGEMT::[PE5-PE2209]
pIZ1759	pGEMT::[PE5-PE2208]
pIZ1812	pKT25::*STM2209*
pIZ1905	pUT18C::*STM2208*
pIZ1906	pUT18C::*STM2209*
pIZ1907	pKT25::*STM2208*

Luria–Bertani (LB) broth was used as liquid medium. Solid LB broth contained agar at 1.5% final concentration. Green plates [17] contained methyl blue (Sigma-Aldrich) instead of aniline blue. The indicator for monitoring ß-galactosidase activity in plate tests was 5-bromo-4-chloro-3-indolyl-ß-D-galactopyranoside (“X-gal”; Sigma-Aldrich, 40 µg/ml). Antibiotics were used at the concentrations described previously [18]. To grow OxyR^−^ strains on LB agar, 75 µl of a 10 mg/ml catalase solution (Sigma-Aldrich) was spread on the surface of the plates.

The oligonucleotides used in this study are listed in Table S1. Targeted gene disruption was achieved using plasmids pKD3, pKD4 and pKD13 [19] and oligonucleotides PS1, PS2 or PS4. Oligonucleotides E1 and E2 were used for allele verification. Antibiotic resistance cassettes introduced during strain construction were excised by recombination with plasmid pCP20 [19]. For the construction of transcriptional and translational *lac* fusions in the *Salmonella* chromosome, FRT sites generated by excision of Km^r^ cassettes were used to integrate either plasmid pCE37 or pCE40 [20]. Addition of 3xFLAG tag to protein-coding DNA sequences was carried out using plasmid pSUB11 as a template [21] and oligonucleotides F2209-5 and F2209-3 (for *STM2209*), and F2208-5 and F2208-3 (for *STM2208*). Transductional crosses using phage P22 HT 105/1 *int201* ([22] and G. Roberts, unpublished data) were used for strain construction operations involving chromosomal markers. The transduction protocol has been described elsewhere [23]. To obtain phage-free isolates, transductants were purified by streaking on green plates. Phage sensitivity was tested by cross-streaking with the clear-plaque mutant P22 H5.

### RNA extraction

RNA was extracted from *S. enterica* stationary phase cultures (OD_600_ ∼3), using the SV total RNA isolation system (Promega) as described at http://www.ifr.ac.uk/safety/microarrays/protocols.html. The quantity and quality of the extracted RNA were determined using an ND-1000 spectrophotometer (NanoDrop Technologies). To diminish genomic DNA contamination, the preparation was treated with DNase I (Turbo DNA free; Applied Biosystems).

### Quantitative reverse transcriptase PCR and calculation of relative expression levels

An aliquot of 0.6 µg of DNase I-treated RNA was used for cDNA synthesis using the High-Capacity cDNA Archive kit (Applied Biosystems). Quantitative reverse transcriptase (RT)-PCR reactions were performed in an Applied Biosystems 7500 Fast Real-Time PCR System. Each reaction was carried out in a total volume of 25 µl on a 96-well optical reaction plate (Applied Biosystems) containing 12.5 µl Power SYBR Green PCR Master Mix (Applied Biosystems), 11.5 µl cDNA (1/10 dilution), and two gene-specific primers (RT2209-5 and RT2209-3 for *STM2209*, RT2208-5 and RT2208-3 for *STM2208*) at a final concentration of 0.2 µM each. Real-time cycling conditions were as follows: (i) 95°C for 10 min and (ii) 40 cycles at 95°C for 15 sec, and 60°C for 1 min. A no-template control was included for each primer set. Melting curve analysis verified that each reaction contained a single PCR product. Gene expression levels were normalized to transcripts of *ompA*, a housekeeping gene that served as an internal control. The Student's *t* test was used to determine if the differences in retrotranscribed mRNA content observed in different backgrounds were statistically significant.

### ß-galactosidase assays

Levels of ß-galactosidase activity were assayed as described previously [24], using the CHCl_3_-sodium dodecyl sulfate permeabilization procedure. The Student's *t* test was used to determine if the differences in ß-galactosidase activities observed in different backgrounds were statistically significant.

### Protein extracts and Western blotting analysis

Total protein extracts were prepared from bacterial cultures grown at 37°C in LB medium until stationary phase (OD_600_ ∼3). Bacterial cells contained in 0.25 ml of culture were collected by centrifugation and suspended in 50 µl of Laemmli sample buffer [1.3% SDS, 10% (v/v) glycerol, 50 mM Tris-HCl, 1.8% β-mercaptoethanol, 0.02% bromophenol blue, pH 6.8]. Proteins were resolved by Tris-Glycine-PAGE using 12% gels (for STM2208) or Tris-Tricine-PAGE 15% gels (for STM2209). Conditions for protein transfer have been described elsewhere [14]. Primary antibodies were anti-Flag M2 monoclonal antibody (1∶5,000, Sigma-Aldrich) and anti-GroEL polyclonal antibody (1∶20,000; Sigma-Aldrich). Goat anti-mouse horseradish peroxidase-conjugated antibody (1∶5,000; Bio-Rad) or goat anti-rabbit horseradish peroxidase-conjugated antibody (1∶20,000; Santa Cruz Biotechnology) was used as secondary antibody. Proteins recognized by the antibodies were visualized by chemoluminescence using the luciferin–luminol reagents (Thermo Scientific).

### Subcellular fractionation

Subcellular fractionation was performed as previously described [25], with some modifications. Briefly, bacteria were grown in LB medium at 37°C and spun down by centrifugation at 15,000× g for 5 min at 4°C, then resuspended twice in cold phosphate-buffered saline (PBS, pH 7.4). The bacterial suspension was either mixed with Laemmli buffer (total protein extract) or disrupted by sonication. Unbroken cells were further removed by low-speed centrifugation (5,000× g, 5 min, 4°C). The supernatant was centrifuged at high speed (100,000× g, 30 min, 4°C) and the new supernatant was recovered as the cytosol fraction. The pellet containing envelope material was suspended in PBS with 0.4% Triton X-100 and incubated for 2 h at 4°C. The sample was centrifuged again (100,000× g, 30 min, 4°C) and divided into the supernatant containing mostly inner membrane proteins and the insoluble fraction corresponding to the outer membrane fraction. An appropiate volume of Laemmli buffer was added to each fraction. After heating (100°C, 5 min) and clearing by centrifugation (15,000× g, 5 min, room temperature), the samples were analyzed for protein content by SDS-PAGE.

### Primer extension

The oligonucleotides PE2209 and PE2208, complementary to internal regions of the genes *STM2209* and *STM2208* respectively, were end-labeled with [^32^P]ATP and annealed to 10 µg of total RNA prepared from *S. enterica* strains bearing plasmids pIZ1758 (constructed using oligonucleotides PE5 and PE2209) and pIZ1759 (constructed with PE5 and PE2208). The end-labeled primer was extended with avian myeloblastosis virus reverse transcriptase (Boehringer Mannheim) under conditions described previously [26]. The products of reverse transcription were analyzed in urea-polyacrylamide gels and visualized using a FLA-5100 Imaging system (Fujifilm).

### Directed construction of point mutations

Mutation of the 4 GATC sites contained in the promoter region of *STM2209-STM2208* was achieved using the QuikChange® Site-Directed Mutagenesis Kit (Stratagene). Briefly, a ∼1.3 Kb fragment of the *STM2209-STM2208* region containing the 4 GATC sites was cloned into the pGEMT plasmid using the oligonucleotides Clo2208-5 and Clo-2208-3. Mutations in every GATC were then introduced using oligonucleotides harboring CATC changes (labeled as DIRnuevo and INVnuevo). The resulting plasmid containing the fragment with 4 CATC sites was then digested with XbaI and SacI, cloned onto the suicide plasmid pDMS197 [27] and propagated in *E. coli* CC118 lambda *pir*. Plasmids derived from pMDS197 were transformed into *E. coli* S17-1 lambda *pir*. The resulting strains were used as donors in matings with *S. enterica* 14028 harboring a Cm^r^ cassette in place of the 4 GATC sites (constructed using oligonucleotides delGATC-PS1 and delGATC-PS2) as recipients. Tc^r^ transconjugants were selected on E plates supplemented with tetracycline. Several Tc^r^ transconjugants were grown in nutrient broth (without NaCl) containing 5% sucrose. Individual tetracycline-sensitive segregants were then screened for cloramphenicol sensitivity and examined for the incorporation of the mutant allelle by Sau3AI digestion and DNA sequencing using external oligonucleotides. Construction of the *oxyR* ^C199S^ mutation was achieved in the same way, using the oligonucleotides ClooxyR-5 and ClooxyR-3 for cloning onto pGEMT, and the oligonucleotides oxyRC199SDIR and oxyRC199SINV for site-directed mutagenesis. A strain with a Cm^r^ cassette in place of the *oxyR* gene (constructed using oligonucleotides deloxyR199-PS1 and deloxyR199-PS2) was used as a recipient in this case.

### Measurement of the efficiency of phage adsorption

The efficiency of phage adsorption was calculated as described by Gabig *et al*. [28]. Briefly, P22 bacteriophages were added to *S. enterica* cells from an LB liquid overnight culture at a multiplicity of infection of 0.1, and the mixture was incubated at 37°C. Samples were taken every 2 min, centrifuged for 1 min at 13,000 rpm in a microcentrifuge, and the supernatant was titrated on the *S. enterica* wild-type strain ATCC 14028. The sample obtained at time zero (a sample taken immediately after addition of bacteriophages to the cell suspension) was considered to correspond to 100% unadsorbed phages, and the remaining numbers were calculated relative to this number. The Student's *t* test was used to determine if the differences in adsorption were statistically significant.

### Electrophoretic visualization of lipopolysaccharide profiles

To investigate lipopolysaccharide (LPS) profiles, bacterial cultures were grown overnight in LB. Bacterial cells were harvested and washed three times with 0.9% NaCl. The O.D._600_ of the washed bacterial suspension was measured to calculate cell concentration. A bacterial mass containing about 3.14×10^8^ cells was pelleted by centrifugation. Treatments applied to the bacterial pellet, electrophoresis of crude bacterial extracts, and silver staining procedures were performed as described by Buendia-Claveria *et al.* [29].

### Calculation of phase transition frequencies

Phase transition rates were estimated as described by Eisenstein [30]. Briefly, a strain harboring an *STM2208*::*lac* fusion was plated on LB + X-gal and colonies displaying an ON or OFF phenotype after 16 h growth at 37°C were selected, resuspended in PBS and respread on new plates. Phase transition frequencies were calculated using the formula *(M/N)/g* where *M* is the number of cells that underwent a phase transition, *N* the total number of cells, and *g* the total number of generations that gave rise to the colony.

### Macrophage infection experiments

The rate of intramacrophage replication after 18 h infection was performed in J774 mouse macrophages as described in [31]. Briefly, macrophages were seeded at a density of 5×10^5^ in 24-well plates and grown in DMEM medium supplemented with 10% (v/v) fetal bovine serum at 37°C, 5% CO2. Bacteria were added to the wells at macrophage-to-bacteria ratio of 1∶10. Phagocytosis was allowed to proceed for 30 min before washing three times with sterile PBS and adding fresh DMEM media supplemented with 20 µg/ml gentamicin. Macrophages were lysed by using 1% Triton X-100, and the number of viable bacteria that survived the gentamicin treatment was determined by subsequent plating onto LB agar plates. The replication rate was determined as the ratio between the number of bacteria at time 18 h and the number of internalized bacteria after 30 min phagocytosis. The Student's *t* test was used to determine if the differences in replication rates observed in different backgrounds were statistically significant.

### Measurement of survival in serum

Survival in guinea pig serum (Sigma-Aldrich) was analyzed as described in [32] with some modifications. Briefly, exponential cultures of *S. enterica* were serially diluted in PBS + 2 mM MgCl_2_ to 2×10^4^ cfu/ml. Guinea pig serum was added to 30% final concentration and the mixtures were incubated at 37°C without shaking. Samples were taken at 30 min intervals by plating on nutrient agar, and viable counts were expressed as a percentage of the initial concentration (% survival). The Student's *t* test was used to determine if the differences in survival to serum observed in different backgrounds were statistically significant.

### Bacterial two-hybrid analysis

The Bacterial Adenylate Cyclase Two-Hybrid (BACTH) system [33] was used to test interaction between two membrane proteins. The *STM2209* and *STM2208* genes were PCR amplified using oligonucleotides pKT25-STM2209-PstI-5 and pKT25-STM2209-BamHI-3 (*STM2209* cloned into pKT25), pUT18C-STM2209-PstI-5 and pKT25-STM2209-BamHI-3 (*STM2209* cloned into pUT18C), pUT18C-STM2208-PstI-5 and pUT18C-STM2208-BamHI-3 (*STM2208* cloned into pUT18C), pKT25-STM2208-PstI-5 and pUT18C-STM2208-BamHI-3 (*STM2208* cloned into pKT25), and cloned onto plasmids pUT18C and pKT25 using the PstI and BamHI sites. Recombinant plasmids carrying *STM2209* and *STM2208* were sequenced using oligonucleotides pKT25-seq5 and pKT25-seq3 and co-transformed into an *E. coli* CyaA^−^ strain (BTH101). Transformants were plated on LB + ampicillin + kanamycin + X-gal medium at 30°C for 30 h. To quantify the interaction between hybrid proteins, bacteria were grown overnight at 30°C in LB + Ap + Km liquid medium supplemented with 0.5 mM isopropyl-ß-D-1-thiogalactopyranoside (IPTG). ß-galactosidase assays were carried out as described above. A level of ß-galactosidase activity at least five fold higher than that measured for vectors alone indicates a positive interaction.

## Results

### *STM2209-STM2208* is a *Salmonella*-specific locus

**Figure 1:**
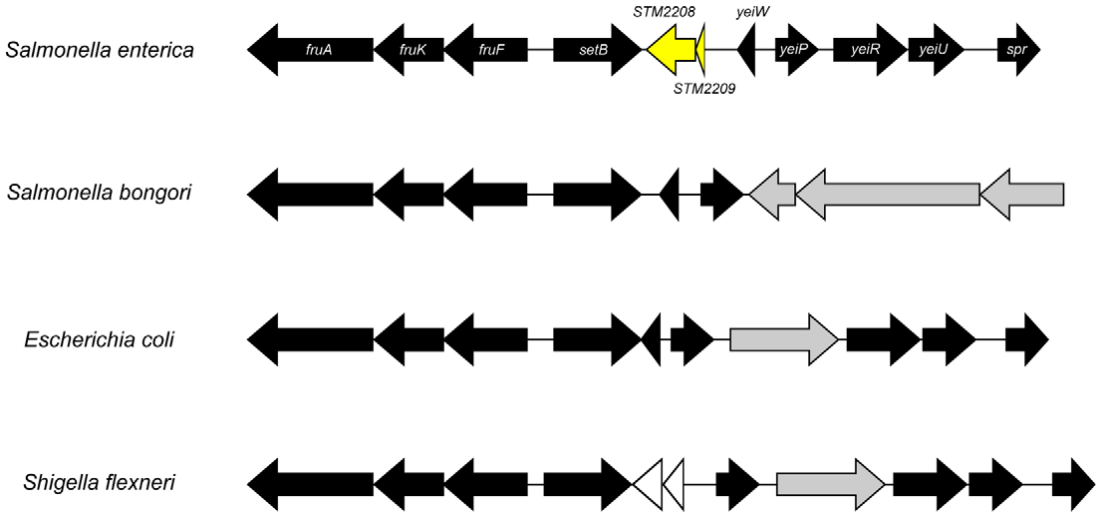
Diagram of the region containing *STM2209-STM2208* on the *Salmonella enterica* chromosome. The homologous regions of *Salmonella bongori, E. coli,* and *Shigella flexneri* are also shown. The *STM2209-STM2208* operon is shown in yellow. Black arrows represent conserved genes. White arrows represent non conserved genes. Grey arrows represent genes found at a different chromosome location on the *S. enterica* chromosome.

S*TM2209* and *STM2208* are contiguous loci annotated as putative protein-coding genes in the chromosome of *Salmonella enterica*. The *STM2209* and *STM2208* ORFs are conserved in *Salmonella enterica* serovar Typhimurium strains ATCC 14028, SL1344, and LT2 (GenBank accession numbers CP001363.1, FQ312003.1 and AE006468.1, respectively), in the vicinity of the sugar transport gene *setB* [34]. The *STM2209* and *STM2208* ORFs are also conserved in other *Salmonella enterica* serovars but not in *Salmonella bongori* nor in the genera *Escherichia* and *Shigella*. Alignment of the predicted amino acid sequences of STM2209 and STM2208 using BLASTP [35] detected no obvious homologs of *STM2209-STM2208* outside *Salmonella enterica*. A diagram of the chromosome region in *Salmonella enterica* and related Enterobacteriaceae is shown in Fig. 1.

Both *STM2209* and *STM2208* have low G+C content (37% for *STM2209* and 38% for *STM2208*) compared to both the average of the region (53%) and that of the *Salmonella enterica* genome (52%) [36]. Because horizontally acquired genes often have distinctive base composition, specifically low G+C content [37], [38], these observations suggest that *STM2209-STM2208* may have been acquired by horizontal gene transfer. The organization of the *STM2209* and *STM2208* ORFs suggests that they may be part of a single transcriptional unit: both coding sequences are on the same DNA strand, and are separated by only one nucleotide. Genome sequence analysis *in silico* predicts that STM2209 may encode a small peptide of 40 amino acids, while STM2208 may be a larger protein product of 221 amino acids. *In silico* analysis of protein structure using the TMHMM transmembrane prediction software [39] predicts the existence of one transmembrane domain in STM2209, and two transmembrane domains in STM2208 (data not shown). *In silico* analysis also indicates that STM2208 shares a domain with proteins belonging to the Wzz superfamily of O-antigen chain length regulators. This family includes proteins involved in lipopolysaccharide biosynthesis that confer a modal distribution of chain length on the O-antigen component of lipopolysaccharide [40]. This domain is also found in bacterial tyrosine kinases [41].

### Expression of the *STM2209-STM2208* locus is regulated by Dam methylation

**Figure 2:**
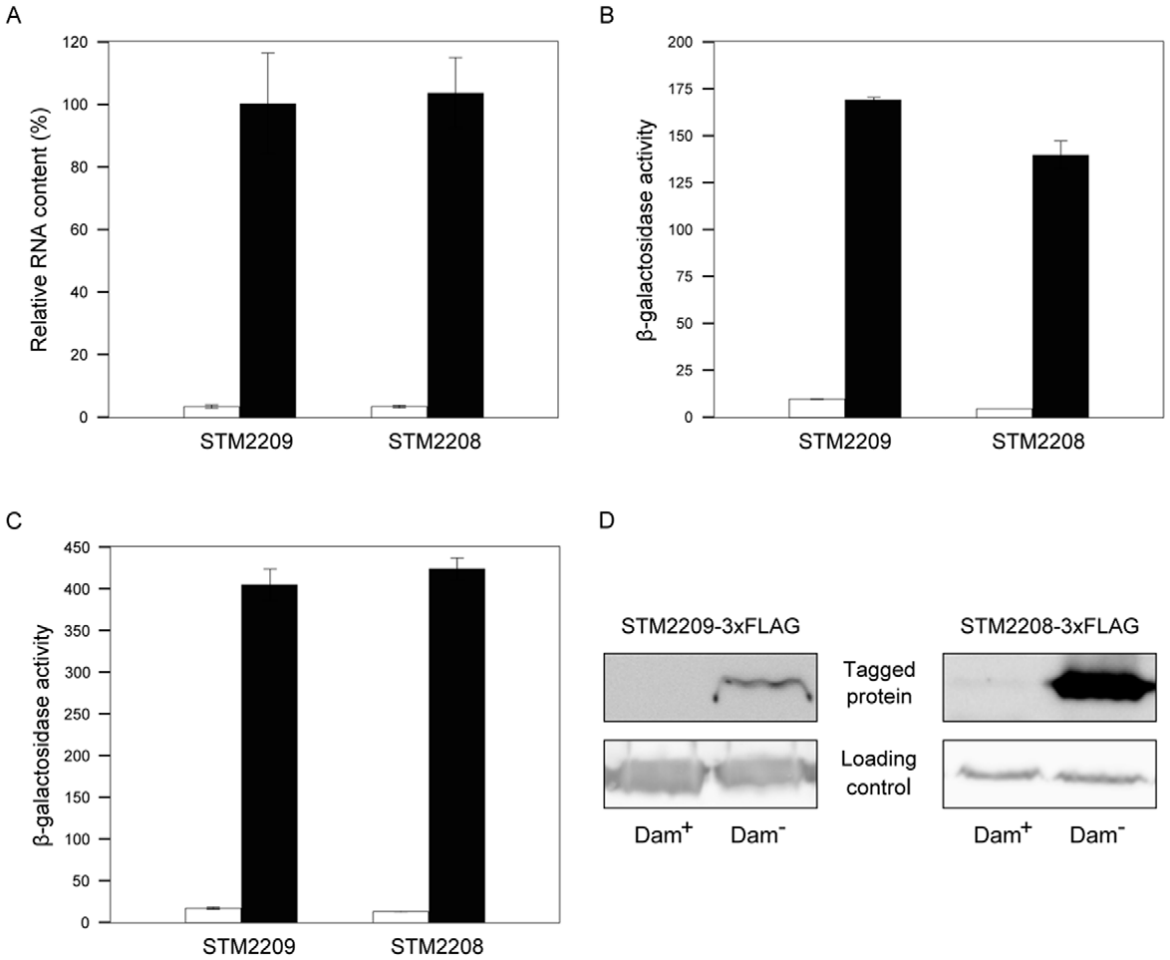
Regulation of *STM2209-STM2208* by Dam methylation. **A.** Levels of *STM2209* and *STM2208* mRNAs, measured by qRT-PCR (Dam^+^: white histograms; Dam^−^: black histograms). Level of *STM2209* mRNA in Dam^−^ background is considered 100%. Values are averages and standard deviations from 7 independent experiments. **B.** ß-galactosidase activity of transcriptional *STM2209::lac* and *STM2208::lac* fusions in Dam^+^ and Dam^−^ backgrounds (white and black histograms, respectively). Values are averages and standard deviations from 3 independent experiments. **C.** ß-galactosidase activities of translational *STM2209::lac* and *STM2208::lac* fusions in Dam^+^ and Dam^−^ backgrounds (white and black histograms, respectively). Values are averages and standard deviations from 3 independent experiments. **D.** Western blot analysis of STM2209-3xFLAG and STM2208-3xFLAG proteins in Dam^+^ and Dam^−^ backgrounds.

A previous study showed that *STM2209* and *STM2208* are expressed at higher levels (13 fold for *STM2209* and 8 fold for *STM2208*) in a *S. enterica* Dam^−^ mutant [16]. These observations suggested that expression of the putative *STM2209-STM2208* transcriptional unit might be repressed by Dam methylation. To confirm Dam-dependent regulation, transcriptional and translational *lac* fusions were constructed in both loci. Protein variants tagged with the 3xFLAG epitope were also constructed. The effect of Dam methylation on *STM2209-STM2208* expression was monitored by ß-galactosidase assays, qRT-PCR, and Western blotting in isogenic Dam^+^ and Dam^−^ strains. Higher level of ß-galactosidase activity, higher amount of retrotranscribed *STM2209-STM2208* mRNA, and increased level of the STM2208-3xFLAG product were detected in the Dam^−^ background (Fig. 2). The STM2209-3xFLAG product was easily detected in a Dam^−^ background but was hardly visible in the Dam^+^ background, presumably due the combined effects of its low level of expression and its small size. STM2209-3xFLAG visualization by Western blotting in a Dam^+^ background was however possible upon longer gel exposure (data not shown). Although the extent of derepression differed slightly depending on the method, expression of *STM2209-STM2208* was significantly higher in a Dam^−^ background in all experiments. These results confirm that Dam methylation represses *STM2209-STM2208*. Furthermore, our ability to detect Dam-dependent regulation with both transcriptional *lac* fusions and qRT-PCR suggests that Dam-dependent regulation of *STM2209-STM2208* may be transcriptional.

### Characterization of the *STM2209-STM2208* transcriptional unit

**Figure 3:**
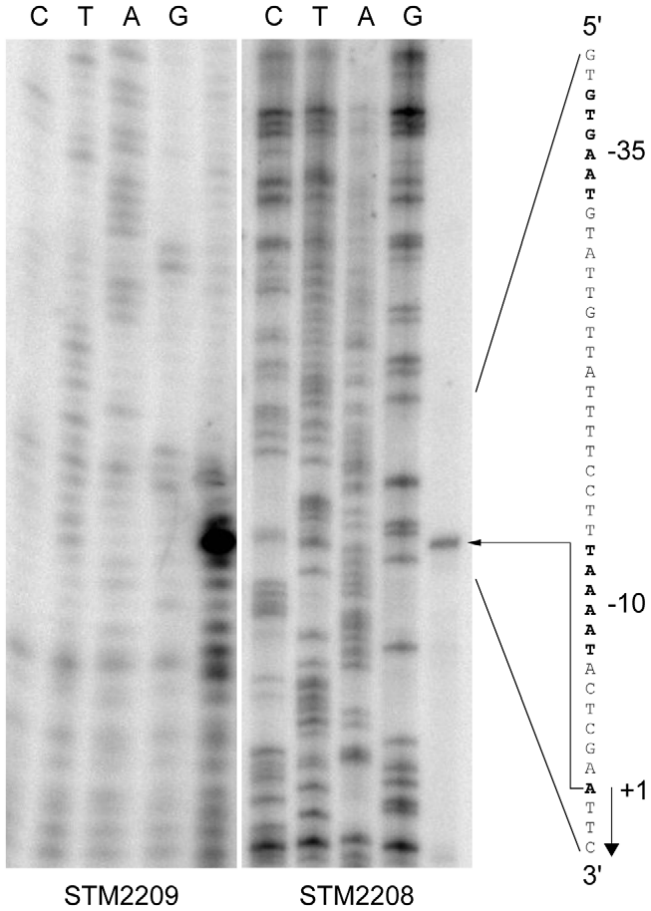
Identification of the transcription initiation site of *STM2209-STM2208* by primer extension. Putative -35 and -10 promoter modules and the +1 site are shown in boldface. The transcription initiation site is indicated by an arrow.

To characterize the *STM2209-STM2208* transcriptional unit, we mapped the 5′ terminus of the putative *STM2209-STM2208* transcript using primer extension (Fig. 3). Because *STM2208* and *STM2209* are expressed at low levels in Dam^+^ *S. enterica* [16], a DNA fragment containing the region upstream *STM2209-STM2208* and part of the coding sequence of *STM2209-STM2208* was cloned on the pGEMT multicopy vector to obtain higher amounts of transcript(s). The resulting plasmids (pIZ1758 and pIZ1759) were introduced in the wild type strain, and two primer extension reactions were performed. One reaction was primed by an oligonucleotide complementary to *STM2209* (PE2209), and the second reaction by an oligonucleotide complementary to *STM2208* (PE2208). Both reactions yielded extension products with identical 3′ ends (Fig. 3), indicating the existence of a single transcription initiation site, six nucleotides upstream the start codon of *STM2209* proposed in the XBASE (http://www.xbase.ac.uk/) and NCBI (http://www.ncbi.nlm.nih.gov/) databases. A DNA sequence reminiscent of a canonical ribosome-binding site is however missing in this putative mRNA organization. For this reason, we propose that translation of *STM2209* may be actually initiated at position +25, 10 nucleotides downstream a putative ribosome binding site (5′ TGTGG 3′). This hypothesis is supported by additional evidence: a translational *lac* fusion constructed upstream +25 proved to be non functional: ß-galactosidase activity was not detected in a Dam^−^ background (data not shown). Altogether, these observations may indicate that STM2209 consists of 34 amino acids and not 40 amino acids as described in the *Salmonella enterica* ATCC 14028 genome annotation.

*In silico* analysis of the DNA sequence upstream of the +1 site identified DNA sequences with features similar to those of canonical, sigma^70^-dependent promoters [42]: (i) a putative −10 module including the motif 5′ TAAAAT 3′, which shows 5/6 matches with the consensus sequence [42]; (ii) a putative spacer, 17 nucleotides long; and (iii) a 5′ GTGAAT 3′ sequence defining a putative −35 module, with 3/6 matches with the consensus sequence [42]. We propose that *STM2209* and *STM2208* are co-transcribed from this promoter, an hypothesis consistent with the observation that the STM2209 and STM2208 products are co-expressed (Fig. 2).

### Identification of OxyR as a regulator of *STM2209-STM2208*

A genetic screen based on the T-POP3 transposon [43] was used to search for positive regulators of *STM2209-STM2208*. For this purpose, a Dam^−^ strain carrying a *lac* translational fusion in *STM2208* (SV5683) was used. This strain forms deep blue colonies on LB supplemented with X-gal. Isolates carrying T-POP3 insertions were selected on LB + tetracycline + kanamycin + X-gal, and white colonies were sought. Only a small white colony was obtained in the screen. Cloning and sequencing of T-POP3 boundaries indicated that T-POP3 had inserted in the *oxyR* gene. OxyR^−^ mutants are severely impaired to form colonies on LB plates [44], thus explaining the small colony size of the isolate. However, the isolate formed large colonies on LB + catalase, a standard procedure that permits colony formation by OxyR^−^ mutants [44]. To confirm that *oxyR* loss-of-function abolished *STM2209-STM2208* expression in a Dam^−^ background, the *oxyR* gene was disrupted using lambda Red recombineering. The resulting strain (SV5925), which carries a null *oxyR* allele, was used in further experiments.

**Figure 4:**
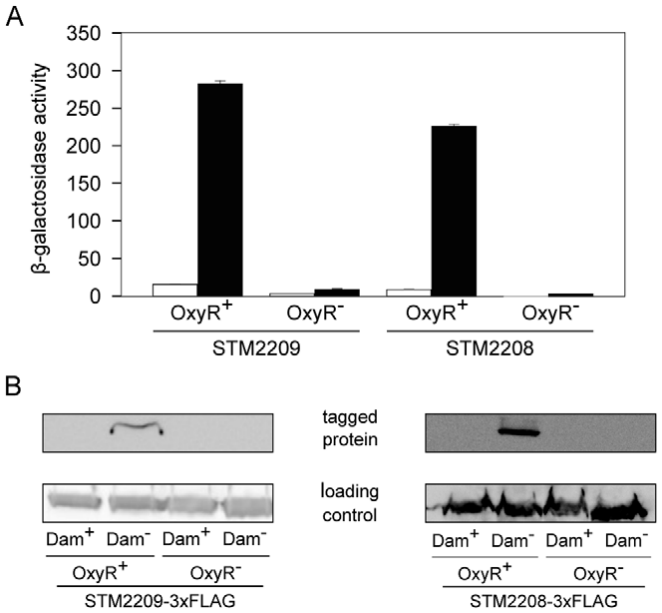
Regulation of *STM2209-STM2208* expression by Dam methylation and OxyR. **A.** Effect of an *oxyR* null mutation on the ß-galactosidase activity of translational *STM2209::lac* and *STM2208::lac* fusions in Dam^+^ and Dam^−^ backgrounds (white and black histograms, respectively). Values are averages and standard deviations from 3 independent experiments. **B.** Western blot analysis of the effect of an *oxyR* null mutation on the levels of STM2209-3xFLAG and STM2208-3xFLAG proteins in Dam^+^ and Dam^−^ backgrounds.

Analyses of ß-galactosidase activity and Western blotting showed that expression of *STM2209-STM2208* is virtually abolished in an OxyR^−^ background (Fig. 4). As above (Fig. 2), high levels of ß-galactosidase and of the STM2209-3xFLAG and STM2208-3xFLAG products were detected in the Dam^−^ background only. These experiments indicate that OxyR is essential for the expression of *STM2209-STM2208*. Interestingly, putative OxyR binding sites are found in the promoter region of *STM2209-STM2208* (see below).

OxyR is a global transcription factor that can sense oxidative stress by direct oxidation. In the oxidized state, OxyR activates the expression of oxidative-stress-responding genes [45]. However, OxyR also acts as a transcriptional regulator irrespective of its oxidative state. In the absence of oxidative stress, OxyR remains mostly in the reduced form due to the reducing environment of the cell [46]. Several observations suggested that the oxidative state of OxyR is not relevant for *STM2209-STM2208* regulation. One was that an H_2_O_2_ concentration sufficient to promote the expression of genes belonging to the classical OxyR regulon (genes activated by oxidative damage) showed no effect on the expression of *STM2209-STM2208* (data not shown). Furthermore, the spacing between the half sites in the putative OxyR binding sites described below is consistent with specific binding of the reduced form of OxyR [47]. To determine the effect of oxidation of OxyR upon *STM2209-STM2208* expression, we constructed a point mutant version of the *oxyR* gene (strain SV6397). The resulting OxyR^C199S^ protein is locked in the reduced form as it cannot form the disulfide bond required for oxidation [46], [47]. Dam^+^ and Dam^−^ strains harboring this mutation showed levels of *STM2209-STM2208* expression similar to those described above for strains carrying the wild type *oxyR* allele (data not shown). These observations suggest that oxidation of OxyR is not necessary for *STM2209-STM2208* expression.

### *STM2209-STM2208* expression undergoes phase variation under the control of Dam methylation and OxyR

In the course of our experiments with strains carrying *STM2209::lac* or *STM2208::lac* fusions in a wild type background, we detected phenotypic heterogeneity when culture aliquots were spread on plates containing X-gal. These strains formed white colonies that later turned pale blue, indicating low expression of *STM2209* and *STM2208*. However, deep blue colonies were also seen, especially on plates that contained high numbers of colonies (e. g., ≥1,000 colonies). Whenever a blue colony was isolated and streaked out for single colonies, a mixture of white and blue colonies was obtained. This observation suggested that *STM2209-STM2208* expression might undergo phase variation, and that switching from OFF to ON might occur at lower frequencies than switching from ON to OFF.

Phase variation frequencies in the *STM2209-STM2208* locus were calculated using the formula *(M/N)/g* where *M* is the number of cells that underwent a phase transition, *N* the total number of cells, and *g* the total number of generations that gave rise to the colony [30]. An *STM2208::lac* translational fusion was used for these experiments. The frequency of OFF→ON transition was estimated to be 6.1±1.7×10^−5^ per cell and generation. The ON→OFF switching rate was around 1,000-fold higher: 3.7±0.1×10^−2^ per cell and generation. Phase variation of *STM2209-STM2208* expression was also unaffected by the oxidation state of OxyR (data not shown).

**Figure 5:**
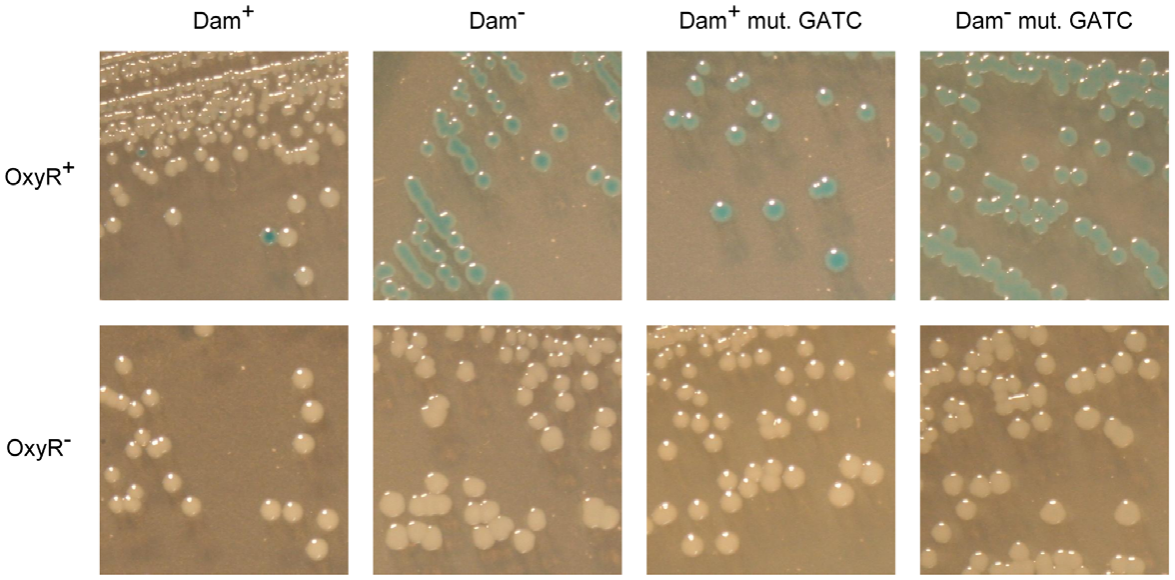
Visual observation of phase variation on LB + X-gal plates in strains carrying an *STM2208*::*lac* fusion in different backgrounds. Strains in the upper row are SV5679 (Dam^+^ OxyR^+^), SV5683 (Dam^−^ OxyR^+^), SV7031 (Dam^+^ OxyR^+^ mut. GATC) and SV7032 (Dam^−^ OxyR^+^ mut. GATC). OxyR^−^ derivatives (SV5989, SV5990, SV7232 and SV7233) are shown in the lower row.

Phase variation was abolished in both Dam^−^ and OxyR^−^ mutants (Fig. 5). Lack of Dam methylation locks *STM2209-STM2208* expression in the ON state, and lack of OxyR locks *STM2209-STM2208* expression in the OFF state. An *oxyR* mutation is epistatic over a *dam* mutation, an observation that may indicate that activation of *STM2209-STM2208* transcription by OxyR is Dam-methylation sensitive. However, both Dam methylation and OxyR are needed to establish phase-variable expression of *STM2209-STM2208*.

### Site-directed mutagenesis of GATC sites upstream the *STM2209-STM2208* promoter abolishes phase variation

**Figure 6:**
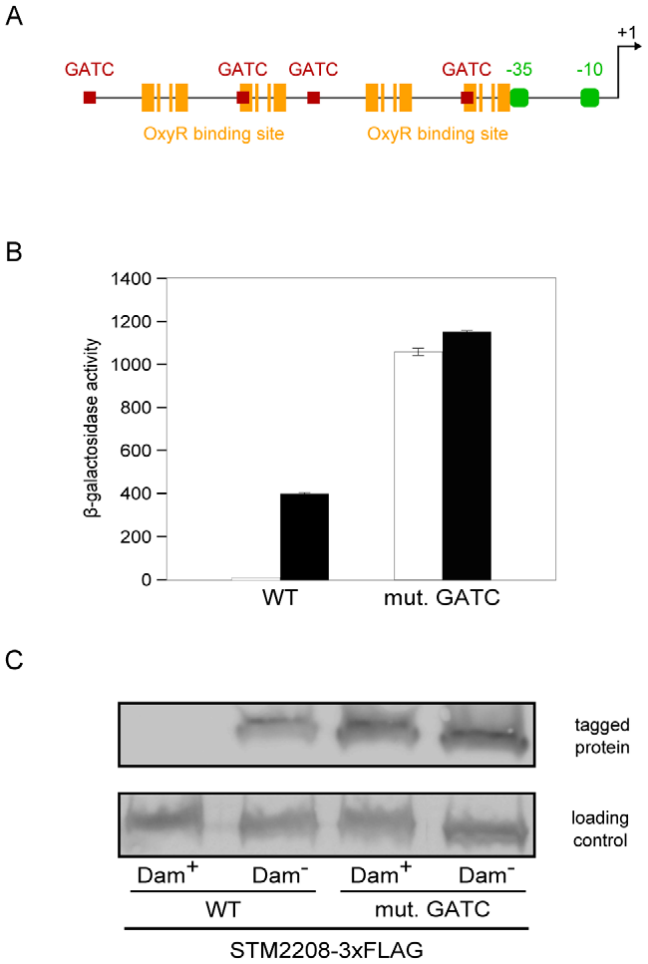
Effect of GATC mutations on *STM2209-STM2208* expression. **A.** Diagram of the promoter region of *STM2209-STM2208*, showing GATC sites (red squares), putative OxyR-binding-sites (orange bars), putative −35 and −10 modules (green boxes) and the transcription initation site (black arrow). **B.** Effect of eliminating the 4 GATC sites upstream the *STM2209-STM2208* promoter on *STM2209-STM2208* expression, monitored by comparing the ß-galactosidase activity of a translational *STM2208::lac* fusion in Dam^+^ and Dam^−^ backgrounds (white and black histograms, respectively). Values are averages and standard deviations from 6 independent experiments. **C.** Effect of eliminating the 4 GATC sites upstream the *STM2209-STM2208* promoter on *STM2209-STM2208* expression, monitored by Western blot analysis of STM2208-3xFLAG levels in different backgrounds.

*In silico* analysis of the DNA sequence upstream the *STM2209-STM2208* promoter revealed the existence of 4 GATC sites arranged in a symmetrical pattern (Fig. 6). In addition, the region contains two putative OxyR binding sites very similar to the consensus sequence [46]. These sites overlap with GATC sites number 2 and 4 respectively (Fig. 6).

Because of the pleiotropy of *dam* mutations, alteration of gene expression in Dam^−^ mutants does not necessarily indicate direct Dam-dependent control [48]. To confirm that Dam methylation directly controls *STM2209-STM2208* expression, the GATC sites present in the promoter region of *STM2209-STM2208* were eliminated by site-directed mutagenesis. If *STM2209-STM2208* repression by Dam methylation depends directly on methylation of the GATC sites within the *STM2209-STM2208* UAS, we reasoned, elimination of the GATCs should lock *STM2209-STM2208* expression in the ON state. To test this prediction, point mutations were engineered to transform the *STM2209-STM2208* 5′GATC3′ sequences to 5′CATC3′ sequences, which are not a substrate for Dam methylase activity (strain SV6401). Furthermore, the four base pair substitutions introduced in the *STM2209-STM2208* UAS do not destroy known critical regions of the OxyR binding sequence [47].

ß-galactosidase activity assays and Western blotting analysis proved that regulation by Dam methylation was abolished when the GATC sites were eliminated (Fig. 6). Expression of *STM2209-STM2208* was ≥2 fold higher in the GATC-less mutant (SV7031) than the Dam^−^ mutant (SV5683) (Fig. 6), but *STM2209-STM2208* expression was locked in the ON state in both strains (Fig. 5). Construction of strain SV6401 thus permitted to analyze the consequences of *STM2209-STM2208* constitutive expression avoiding the pleiotropic effects of *dam* mutations (see below).

### The STM2209 and STM2208 gene products are proteins located in the inner (cytoplasmic) membrane of *Salmonella enterica*

**Figure 7:**
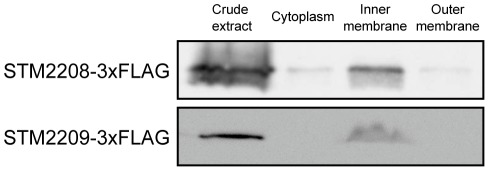
Distribution of STM2209 and STM2208 proteins tagged with a 3xFLAG epitope in subcellular fractions of *S. enterica* serovar Typhimurium. Anti-FLAG Western hybridization is shown for three fractions: cytoplasm, inner membrane, and outer membrane. The volume loaded for all fractions was normalized to the same number of bacteria (7×10^7^ c.f.u.).

The subcellular location of STM2209 and STM2208 was investigated using 3xFLAG-tagged variants. Electrophoretic separation of cell fractions (cytosol, cytoplasmic membrane and outer membrane) was performed, and Western analysis of the separated protein preparations was carried out with a commercial anti-FLAG antibody. The results unambiguously showed that STM2209 and STM2208 are located in the *S. enterica* inner (cytoplasmic) membrane (Fig. 7).

### Evidence for interaction between STM2209 and STM2208 in the *Salmonella* cytoplasmic membrane

**Figure 8:**
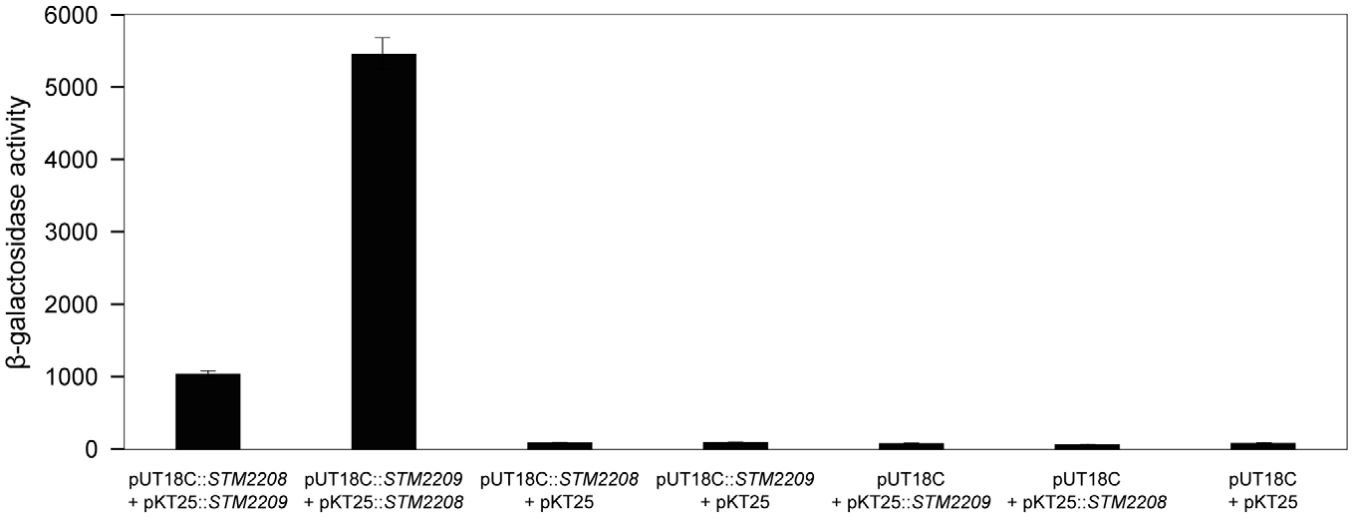
Analysis of the *in vivo* interaction between STM2209 and STM2208 using the BACTH system. The *E. coli* BTH101 strain was co-transformed with plasmids encoding fusion proteins or empty. The basal level of ß-galactosidase activity measured with empty vectors was approximately 90 Miller units. Values are averages and standard deviations from 3 independent experiments.

STM2209 may represent a novel example of a membrane peptide, an emerging class of functional molecules [49]. Because certain membrane peptides have been shown to interact with membrane protein partners, we investigated whether STM2209 interacts with the inner-membrane protein STM2208. To test interaction between STM2209 and STM2208 *in vivo*, we used the Bacterial Adenylate Cyclase Two-Hybrid (BACTH) assay, a procedure that permits the detection of specific interactions between inner membrane proteins [50]. *STM2209* and *STM2208* were independently cloned on plasmids pUT18C and pKT25. Four plasmid constructs were obtained (pUT18C-*STM2209*, pKT25-*STM2209*, pUT18C-*STM2208*, and pKT25-*STM2208*), and their interaction was tested in an *E. coli* CyaA^−^ mutant (BTH101). Functional complementation was determined by measuring ß-galactosidase activity. High levels of ß-galactosidase activity were obtained with both plasmid pairs, compared with the basal activities of the plasmid vectors or with the activity of one fusion protein only (Fig. 8). These results suggest that STM2209 and STM2208 may interact indeed.

### Constitutive expression of *STM2209-STM2208* reduces P22 adsorption to *S. enterica*

**Figure 9:**
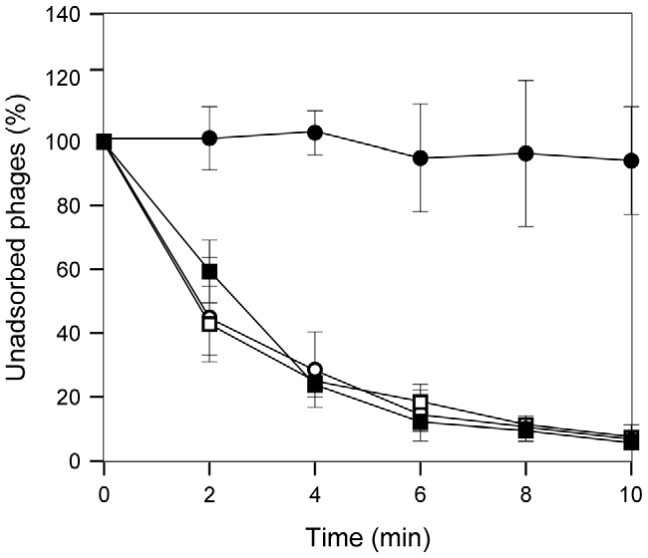
Effect of constitutive expression of *STM2209-STM2208* on adsorption of bacteriophage P22 to *S. enterica*. The efficiency of P22 attachment to *S. enterica* is shown as the percentage of non adsorbed phages relative to the initial number. Strains are represented by black squares (wild type), white squares (SV6013, Δ*STM2209-STM2208*), black circles (SV6401, mut. GATC) and white circles (SV6976, mut. GATC Δ*STM2209-STM2208*). Values are averages and standard deviations from 6 independent experiments.

During strain construction experiments by P22 HT transduction, we obtained reduced numbers of transductants whenever the strain that constitutively expresses *STM2209-STM2208* (SV6401) was used as a recipient. This observation, combined with the fact that STM2209 and STM2208 are components of the cell envelope, raised the possibility that constitutive synthesis of STM2209 and STM2208 might impair adsorption of bacteriophage P22. To test this hypothesis, we compared the kinetics of P22 adsorption to the wild type strain, to a strain that constitutively expresses *STM2209-STM2208* (SV6401), and to a strain that harbors a deletion of *STM2209-STM2208* (SV6013). Suspensions of P22 bacteriophage and *S. enterica* were mixed, and samples were taken every two minutes, and centrifuged. The supernatant was subsequently titrated to monitor the presence of unattached phages (Fig. 9). Adsorption of P22 to *S. enterica* cells was found to be severely impaired in the strain that constitutively expressed *STM2209-STM2208* (SV6401), which proved to be largely refractory to phage P22 attachment. In contrast, P22 adsorption remained unaltered in a strain carrying a *STM2209-STM2208* deletion (SV6013) regardless of the presence of the mutated GATCs (SV6976). These experiments suggest that phase variation of *STM2209-STM2208* may split clonal populations of *S. enterica* into two subpopulations, one of which is P22-sensitive while the other is P22-resistant.

### Constitutive expression of *STM2209-STM2208* alters chain length distribution in the lipopolysaccharide O-antigen of *S. enterica*

**Figure 10:**
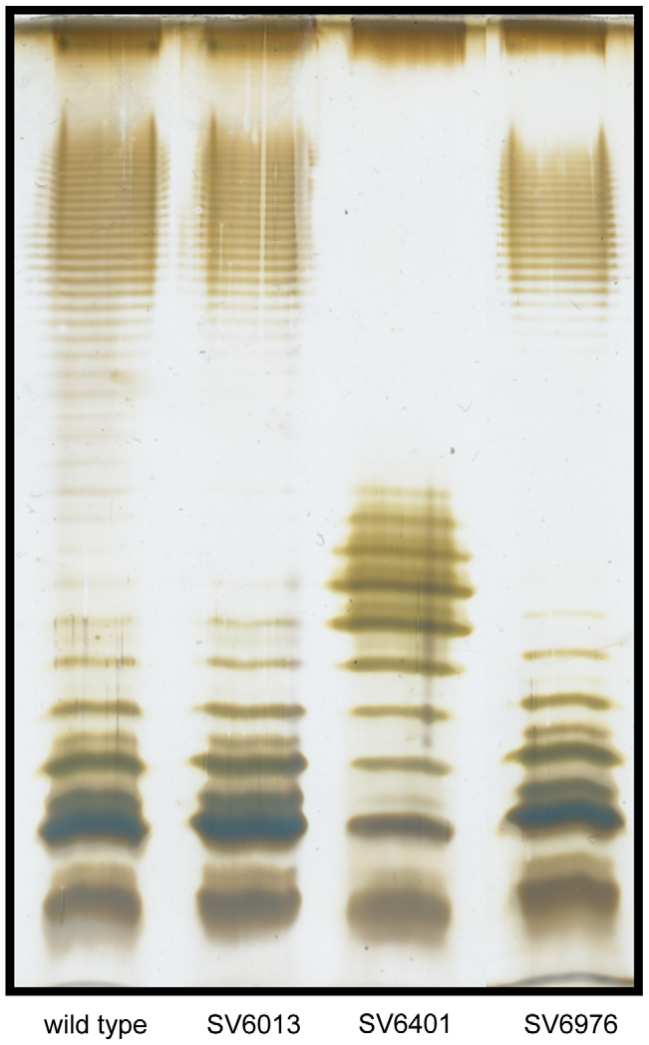
Lipopolysaccharide profiles of the wild type strain (lane 1), SV6013 (Δ*STM2209-STM2208*) (lane 2), SV6401 (mut. GATC) (lane 3) and SV6976 (mut. GATC Δ*STM2209-STM2208*) (lane 4), as observed by electrophoresis and silver staining.

Because phage P22 is known to attatch to the LPS of *Salmonella enterica* to initiate infection [51], we examined whether the strain that constitutively expresses *STM2209-STM2208* (SV6401) showed LPS alterations. Migration of the LPS in polyacrylamide gel is known to be affected by the number and size of repeating oligosaccharide units in long-chain LPS, such that bands in the profile represent progressively larger concatemers of the repeating oligosaccharide units [52]. Comparison of the LPS profiles in strain SV6401 and the wild type revealed drastic alterations in the length of O-antigen chains (Fig. 10). Wild type *Salmonella* LPS shows a bimodal distribution typical of many Enterobacteriaceae, with higher amounts of bands with 16–35 and >100 repeats [40], [53]–[55]. Strain SV6401 showed a unimodal distribution, with bands concentrated in the 3–8 repeat range. This short and homogeneous LPS might well explain reduced phage P22 attachment. No alteration of the LPS profile was detected in a strain carrying a *STM2209-STM2208* deletion (SV6013), in agreement with its ability to permit a normal level of P22 adsorption (Fig. 9). The main conclusion from these experiments was that expression of *STM2209-STM2208* alters O-antigen chain length.

### Constitutive expression of *STM2209-STM2208* reduces *S. enterica* resistance to guinea pig serum

**Figure 11:**
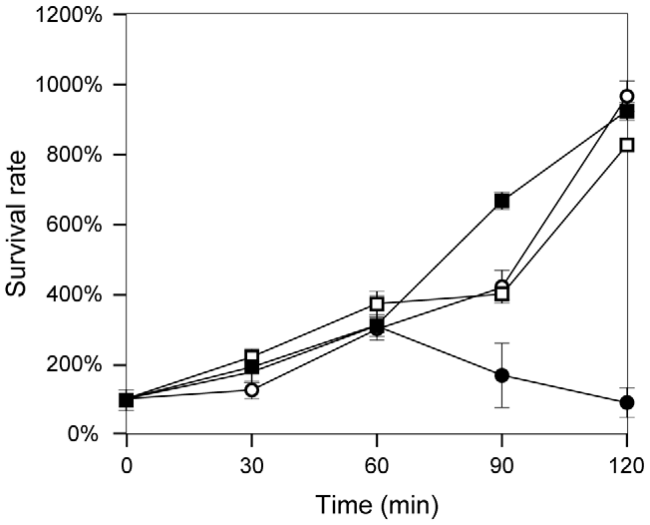
Survival in presence of 30% guinea pig serum. Strains are represented by black squares (wild type), white squares (SV6013, Δ*STM2209-STM2208*), black circles (SV6401, mut. GATC) and white circles (SV6976, mut. GATC Δ*STM2209-STM2208*). Values are averages and standard deviations from 5 independent experiments.

O-antigen chain length has been described to be crucial for serum resistance in *Salmonella* [54], [56]–[59]. Survival in serum was analyzed by treating exponentially growing cells with 30% non-immune guinea pig serum. Constitutive expression of *STM2209-STM2208* caused increased killing by serum (Fig. 11). This is likely to be complement-mediated, since heat-inactivated serum did not impair growth of strain SV6401 (data not shown).

### Constitutive expression of *STM2209-STM2208* reduces *S. enterica* proliferation in macrophages

**Figure 12:**
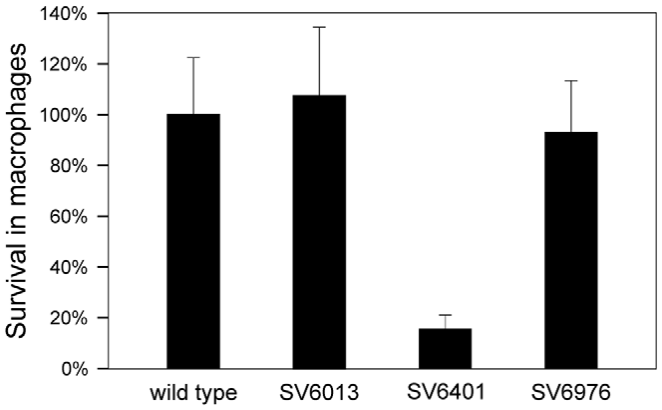
Rate of intramacrophage proliferation for the wild type strain (lane 1), SV6013 (Δ*STM2209-STM2208*) (lane 2), SV6401 (mut. GATC) (lane 3) and SV6976 (mut. GATC Δ*STM2209-STM2208*) (lane 4). Values are averages and standard deviations from 3 independent experiments.

Additional screens and phenotypic assays were performed in search for functions of *STM2209-STM2208* phase variation besides the formation of a P22-resistant subpopulation with reduced resistance to serum. The trials included: (i) growth in various media at different temperatures and different osmolarities; (ii) resistance to acidic pH, cationic peptides, bile, and hydrogen peroxide; (iii) motility; (iv) biofilm formation; (v) and invasion of and proliferation in epithelial and macrophage cell lines. Most trials did not show differences associated either to loss or constitutive expression of *STM2209-STM2208*. A remarkable exception was that constitutive expression of *STM2209-STM2208* impaired intracellular proliferation within macrophages (Fig. 12). On the other hand, a strain carrying a *STM2209-STM2208* deletion showed intramacrophage proliferation at a level similar level to that of the wild-type strain. These observations suggest that repression of *STM2209-STM2208* expression may be required to permit *Salmonella* proliferation within macrophages. However, a nonproliferating *S. enterica* population may be also generated by switching *STM2209-STM2208* to the ON state.

## Discussion

*STM2209* and *STM2208*, hitherto annotated as putative genes of unknown function in the genome of *Salmonella enterica* serovar Typhimurium, are absent in *Salmonella bongori* and in other species of enteric bacteria (Fig. 1). This assortment, combined with G+C content lower than the core *Salmonella* genome (38% *vs.* 52%, approximately), suggests acquisition by horizontal transfer.

*STM2209* and *STM2208* are part of a single transcriptional unit, and are transcribed from a promoter upstream *STM2209* (Fig. 3). The *STM2209* gene product is a small hydrophobic peptide (putatively, 34 amino acids) while *STM2208* encodes a larger hydrophobic protein (putatively, 221 amino acids). Both STM2209 and STM2208 are located in the cytoplasmic membrane (Fig. 7). Certain structural features of STM2209 and STM2208 are reminiscent of those found in interacting peptide-protein pairs located in the bacterial cytoplasmic membrane [49]. For instance, the putative transmembrane domain of STM2209 and the putative N-terminus-proximal transmembrane domain of STM2208 are rich in phenylalanine and share additional amino acid sequence features. STM2209 and STM2208, however, lack common packing motifs described elsewhere for transmembrane-helix interactions, such as GxxxG, Ala-coil or motifs of serine and threonine [60]–[62]. Small regulatory peptides often interact with larger proteins encoded in the same transcriptional unit, modulating their activity or stability [49]. This study presents evidence that STM2209 and STM2208 interact indeed (Fig. 8). The functional significance of STM2209-STM2208 interaction remains unknown; a tentative analogy with other peptide-protein pairs [49], [63] permits the speculation that the STM2209 peptide might modulate the function of the STM2208 protein or act as a subunit in a larger complex.

Expression of the *STM2209-STM2208* locus is subjected to phase variation (Fig. 5), and the OFF→ON switching frequency in LB medium is 3 orders of magnitude lower than ON→OFF switching (6.1×10^−5^ *vs.* 3.7×10^−2^ per cell and generation). Skewed frequencies of switching are also found in other phase variation loci: for instance, in the *E. coli pap* operon, the OFF→ON switching frequency is 5.54×10^−4^ per cell and generation, while the ON→OFF switching frequency is 2.34×10^−2^ per cell and generation [64]. Hence, like in *pap*, the subpopulation of cells that express *STM2209-STM2208* in LB is smaller than the population of cells that do not express *STM2209-STM2208*. However, the switching frequencies detected under laboratory conditions can be different from those occurring in natural environments [8], [65]. In the *pap* operon, for instance, the switching frequencies are skewed by environmental inputs involving global regulators like Crp and H-NS and the stress-responsive system CpxRA [66]–[68].

Lack of Dam methylation locks *STM2209-STM2208* in the ON state (Fig. 5), thus explaining why *STM2209-STM2208* was initially considered a locus repressed by Dam methylation [16]. Dam methylation has been previously shown to control phase variation systems along with a variety of transcriptional regulators [8]. However, Dam methylation can also regulate gene expression indirectly, either as a consequence of lack of DNA mismatch repair or by controlling expression of postranscriptional regulators [48], [69]. In the case of *STM2209-STM2208*, the observation that site-directed mutagenesis of GATC sites located upstream the *STM2209-STM2208* promoter locks expression in the ON state (Fig. 6) provides preliminary evidence that Dam methylation may regulate *STM2209-STM2208* transcription. Evidence that *STM2209-STM2208* is a new locus under the control of a Dam-sensitive transcriptional regulator is further supported by the identification of the LysR-like factor OxyR as a positive regulator of *STM2209-STM2208* expression (Fig. 4). OxyR is a well known transcriptional regulator [45], and has been previously shown to control phase variation of other Dam methylation-sensitive loci: the *E. coli agn43* gene [70], [71] and the P22 *gtr* operon [4]. Unlike *agn43*, which is repressed by OxyR [70], and *gtr*, which is both activated and repressed by OxyR [4], *STM2209-STM2208* is under positive control by OxyR (Fig. 4). Like in *agn43* and in *gtr*, however, the oxidation state of OxyR is irrelevant for control of *STM2209-STM2208* expression.

When *STM2209-STM2208* expression is locked in the ON state, *Salmonella* cells become resistant to phage P22 (Fig. 9), presumably by alteration of O-antigen chain length in the lipopolysaccharide (Fig. 10). Hence, phase variation of *STM2209-STM2208* expression in wild type populations of *Salmonella* can be expected to generate a subpopulation of P22-resistant cells. Resistance might be potentially extended to other *Salmonella*-specific lambdoid bacteriophages [72]. Phase variation in mechanisms of defense against bacteriophage infection has been previously described [6]. A phase variation system that controls *Salmonella* lipopolysaccharide modification has been also described in phage P22 [4]. However, to our knowledge, *STM2209-STM2208* may be the first example of a phase variation system that confers phage resistance through alteration of O-antigen chain length.

O-antigen alteration may be also the cause of two infection-related traits associated to *STM2209-STM2208* expression. One is increased sensitivity to serum (Fig. 11), which may be explained by the involvement of O-antigen chain length in serum resistance [32], [54], [56], [58]. Reduced capacity to proliferate in macrophages (Fig. 12) could also be attributed to modification of the structure of LPS [73], [74], although the relevance of O-antigen chain length in the *Salmonella*-macrophage interaction has been questioned [75], [76]. On the other hand, LPS-containing outer membrane vesicles have been shown to mediate delivery of *Salmonella* virulence effectors to macrophages [77], suggesting that constitutive synthesis of STM2209 and STM2208 might impair the secretion process. Current evidence suggests that diversity in the structure and distribution of O-antigen length permits a balance between resistance to antimicrobial compounds and the ability to interact with different cell types [75]. Indeed, it has been described that O-antigen length is reduced upon growth inside murine macrophages [78] in a way reminiscent of the effect of constitutive expression of *STM2209-STM2208* (Fig. 10). Interestingly, expression of *STM2209-STM2208* is upregulated inside epithelial cells and macrophages [79].

STM2208 displays features typical of Gram-negative O-antigen chain length regulators such as Wzz_ST_ (16–35 repeats) and Wzz_fepE_ (>100 repeats): a common protein structure consisting of two transmembrane domains and a hydrophilic periplasmic domain, relative richness in proline residues in the second transmembrane segment [80], and a particular set of conserved amino acid residues near the N-terminal end [81]. STM2208 lacks, however, a predicted coiled-coil periplasmic domain typical of many O-antigen chain length regulators [80]. However, other O-antigen chain length regulators show little or no potential for coiled-coil formation. Furthermore, there is a correlation between coiled-coil potential of the periplasmic domain and the modal length conferred on the LPS O-antigen chains [80]. Because constitutive expression of *STM2209-STM2208* leads to short modal length of the O-antigen (Fig. 10), lack of coiled-coil potential is not surprising.

Formation of a phage-resistant subpopulation upon *STM2209-STM2208* phase variation may have obvious selective value. In contrast, the potential advantage of forming a less virulent bacterial subpopulation may be at the first sight intriguing. However, subpopulation formation has been described at several stages of host colonization by *Salmonella*, and a tentative interpretation is that reduction or arrest of bacterial growth is part of a stealthy strategy that increases the chances of successful infection. For instance, bistability in the synthesis of flagellin helps *Salmonella* to evade the host caspase-1 inflammatory response [3]. Another example is found upon *Salmonella* entry into macrophages: the population splits into two subpopulations, one of which replicates while the other enters a dormant-like state [82]. It has also been suggested that a successful infection strategy might involve the sacrifice of a fraction of the total population [83]. It might be argued that the rate of *STM2209-STM2208* switching to the ON state (approximately, 6×10^−5^ per cell and generation) may be too low to produce a bacterial subpopulation of relevant size in animal tissues, especially in macrophages which typically host very low numbers of *Salmonella* cells [84]. However, as discussed above, the switching rates observed in the laboratory may not apply to other growth conditions [1]. Actually, the introduction of deterministic elements in stochastic gene regulation may be a common feature of phase variation systems [65]. Phase variation of *STM2209-STM2208* might thus occur at different rates in different environments. Formation of a phage-resistant subpopulation, however, can be expected to have selective value regardless of the subpopulation size.

We propose that the *STM2209-STM2208* locus is renamed *opv* (for O-antigen phase variation) so that the *STM2209* gene is henceforth known as *opvA*, and the *STM2208* gene as *opvB*.

## Supporting Information

Table S1 Oligonucleotides.
(DOC)

## References

[1] van der Woude MW, Bäumler AJ (2004). Phase and antigenic variation in bacteria..

[2] van der Woude MW (2011). Phase variation: how to create and coordinate population diversity.. Curr Opin Microbiol.

[3] Stewart MK, Cummings LA, Johnson ML, Berezow AB, Cookson BT (2011). Regulation of phenotypic heterogeneity permits Salmonella evasion of the host caspase-1 inflammatory response.. Proc Natl Acad Sci U S A.

[4] Broadbent SE, Davies MR, van der Woude MW (2010). Phase variation controls expression of Salmonella lipopolysaccharide modification genes by a DNA methylation-dependent mechanism.. Mol Microbiol.

[5] Cummings CA, Bootsma HJ, Relman DA, Miller JF (2006). Species- and strain-specific control of a complex, flexible regulon by Bordetella BvgAS.. J Bacteriol.

[6] Hoskisson PA, Smith MC (2007). Hypervariation and phase variation in the bacteriophage ‘resistome’.. Curr Opin Microbiol.

[7] Srikhanta YN, Fox KL, Jennings MP (2010). The phasevarion: phase variation of type III DNA methyltransferases controls coordinated switching in multiple genes.. Nat Rev Microbiol.

[8] Casadesus J, Low D (2006). Epigenetic gene regulation in the bacterial world.. Microbiol Mol Biol Rev.

[9] Low DA, Casadesus J (2008). Clocks and switches: bacterial gene regulation by DNA adenine methylation.. Curr Opin Microbiol.

[10] van der Woude M, Braaten B, Low D (1996). Epigenetic phase variation of the *pap* operon in *Escherichia coli*.. Trends Microbiol.

[11] Henderson IR, Owen P (1999). The major phase-variable outer membrane protein of *Escherichia coli* structurally resembles the immunoglobulin A1 protease class of exported protein and is regulated by a novel mechanism involving Dam and OxyR.. J Bacteriol.

[12] Crost C, Garrivier A, Harel J, Martin C (2003). Leucine-responsive regulatory protein-mediated repression of *clp* (encoding CS31A) expression by L-leucine and L-alanine in *Escherichia coli*.. J Bacteriol.

[13] Nicholson B, Low D (2000). DNA methylation-dependent regulation of *pef* expression in *Salmonella typhimurium*.. Mol Microbiol.

[14] Jakomin M, Chessa D, Bäumler AJ, Casadesus J (2008). Regulation of the *Salmonella enterica std* fimbrial operon by DNA adenine methylation, SeqA, and HdfR.. J Bacteriol.

[15] McClelland M, Sanderson KE, Spieth J, Clifton SW, Latreille P (2001). Complete genome sequence of *Salmonella enterica* serovar Typhimurium LT2.. Nature.

[16] Balbontin R, Rowley G, Pucciarelli MG, Lopez-Garrido J, Wormstone Y (2006). DNA adenine methylation regulates virulence gene expression in *Salmonella enterica* serovar Typhimurium.. J Bacteriol.

[17] Chan RK, Botstein D, Watanabe T, Ogata Y (1972). Specialized transduction of tetracycline resistance by phage P22 in *Salmonella typhimurium*. II. Properties of a high-frequency-transducing lysate.. Virology.

[18] Torreblanca J, Marques S, Casadesus J (1999). Synthesis of FinP RNA by plasmids F and pSLT is regulated by DNA adenine methylation.. Genetics.

[19] Datsenko KA, Wanner BL (2000). One-step inactivation of chromosomal genes in *Escherichia coli* K-12 using PCR products.. Proc Natl Acad Sci U S A.

[20] Ellermeier CD, Janakiraman A, Slauch JM (2002). Construction of targeted single copy lac fusions using lambda Red and FLP-mediated site-specific recombination in bacteria.. Gene.

[21] Uzzau S, Figueroa-Bossi N, Rubino S, Bossi L (2001). Epitope tagging of chromosomal genes in Salmonella.. Proc Natl Acad Sci U S A.

[22] Schmieger H (1972). Phage P22-mutants with increased or decreased transduction abilities.. Mol Gen Genet.

[23] Garzon A, Cano DA, Casadesus J (1995). Role of Erf recombinase in P22-mediated plasmid transduction.. Genetics.

[24] Miller JH, Harbor ColdSpring (1972). Experiments in molecular genetics.. N.Y.: Cold Spring Harbor Laboratory.

[25] Pucciarelli MG, Prieto AI, Casadesus J, Garcia-del Portillo F (2002). Envelope instability in DNA adenine methylase mutants of *Salmonella enterica*.. Microbiology.

[26] Marques S, Ramos JL, Timmis KN (1993). Analysis of the mRNA structure of the *Pseudomonas putida* TOL meta fission pathway operon around the transcription initiation point, the *xylTE* and the *xylFJ* regions.. Biochim Biophys Acta.

[27] Hautefort I, Proenca MJ, Hinton JC (2003). Single-copy green fluorescent protein gene fusions allow accurate measurement of Salmonella gene expression in vitro and during infection of mammalian cells.. Appl Environ Microbiol.

[28] Gabig M, Herman-Antosiewicz A, Kwiatkowska M, Los M, Thomas MS (2002). The cell surface protein Ag43 facilitates phage infection of *Escherichia coli* in the presence of bile salts and carbohydrates.. Microbiology.

[29] Buendia-Claveria AM, Moussaid A, Ollero FJ, Vinardell JM, Torres A (2003). A *purL* mutant of *Sinorhizobium fredii* HH103 is symbiotically defective and altered in its lipopolysaccharide.. Microbiology.

[30] Eisenstein BI (1981). Phase variation of type 1 fimbriae in *Escherichia coli* is under transcriptional control.. Science.

[31] Rang C, Alix E, Felix C, Heitz A, Tasse L (2007). Dual role of the MgtC virulence factor in host and non-host environments.. Mol Microbiol.

[32] Murray GL, Attridge SR, Morona R (2005). Inducible serum resistance in *Salmonella typhimurium* is dependent on *wzz(fepE)*-regulated very long O antigen chains.. Microbes Infect.

[33] Karimova G, Fayolle C, Gmira S, Ullmann A, Leclerc C (1998). Charge-dependent translocation of *Bordetella pertussis* adenylate cyclase toxin into eukaryotic cells: implication for the in vivo delivery of CD8(+) T cell epitopes into antigen-presenting cells.. Proc Natl Acad Sci U S A.

[34] Liu JY, Miller PF, Willard J, Olson ER (1999). Functional and biochemical characterization of *Escherichia coli* sugar efflux transporters.. J Biol Chem.

[35] Altschul SF, Madden TL, Schaffer AA, Zhang J, Zhang Z (1997). Gapped BLAST and PSI-BLAST: a new generation of protein database search programs.. Nucleic Acids Res.

[36] Navarre WW, Porwollik S, Wang Y, McClelland M, Rosen H (2006). Selective silencing of foreign DNA with low GC content by the H-NS protein in Salmonella.. Science.

[37] Medigue C, Rouxel T, Vigier P, Henaut A, Danchin A (1991). Evidence for horizontal gene transfer in *Escherichia coli* speciation.. J Mol Biol.

[38] Daubin V, Ochman H (2004). Start-up entities in the origin of new genes.. Curr Opin Genet Dev.

[39] Krogh A, Larsson B, von Heijne G, Sonnhammer EL (2001). Predicting transmembrane protein topology with a hidden Markov model: application to complete genomes.. J Mol Biol.

[40] Morona R, van den Bosch L, Manning PA (1995). Molecular, genetic, and topological characterization of O-antigen chain length regulation in *Shigella flexneri*.. J Bacteriol.

[41] Marchler-Bauer A, Lu S, Anderson JB, Chitsaz F, Derbyshire MK (2011). CDD: a Conserved Domain Database for the functional annotation of proteins.. Nucleic Acids Res.

[42] Huerta AM, Collado-Vides J (2003). Sigma70 promoters in *Escherichia coli*: specific transcription in dense regions of overlapping promoter-like signals.. J Mol Biol.

[43] Rappleye CA, Roth JR (1997). A Tn*10* derivative (T-POP) for isolation of insertions with conditional (tetracycline-dependent) phenotypes.. J Bacteriol.

[44] Hassett DJ, Alsabbagh E, Parvatiyar K, Howell ML, Wilmott RW (2000). A protease-resistant catalase, KatA, released upon cell lysis during stationary phase is essential for aerobic survival of a *Pseudomonas aeruginosa oxyR* mutant at low cell densities.. J Bacteriol.

[45] Storz G, Tartaglia LA, Ames BN (1990). The OxyR regulon.. Antonie Van Leeuwenhoek.

[46] Kullik I, Toledano MB, Tartaglia LA, Storz G (1995). Mutational analysis of the redox-sensitive transcriptional regulator OxyR: regions important for oxidation and transcriptional activation.. J Bacteriol.

[47] Toledano MB, Kullik I, Trinh F, Baird PT, Schneider TD (1994). Redox-dependent shift of OxyR-DNA contacts along an extended DNA-binding site: a mechanism for differential promoter selection.. Cell.

[48] Marinus MG, Casadesus J (2009). Roles of DNA adenine methylation in host-pathogen interactions: mismatch repair, transcriptional regulation, and more.. FEMS Microbiol Rev.

[49] Alix E, Blanc-Potard AB (2009). Hydrophobic peptides: novel regulators within bacterial membrane.. Mol Microbiol.

[50] Karimova G, Dautin N, Ladant D (2005). Interaction network among *Escherichia coli* membrane proteins involved in cell division as revealed by bacterial two-hybrid analysis.. J Bacteriol.

[51] Wright A, Kanegasaki S (1971). Molecular aspects of lipopolysaccharides.. Physiol Rev.

[52] Wu T, McCandlish AC, Gronenberg LS, Chng SS, Silhavy TJ (2006). Identification of a protein complex that assembles lipopolysaccharide in the outer membrane of *Escherichia coli*.. Proc Natl Acad Sci U S A.

[53] Raetz CR, Whitfield C (2002). Lipopolysaccharide endotoxins.. Annu Rev Biochem.

[54] Murray GL, Attridge SR, Morona R (2003). Regulation of *Salmonella typhimurium* lipopolysaccharide O antigen chain length is required for virulence: identification of FepE as a second Wzz.. Mol Microbiol.

[55] Peterson AA, McGroarty EJ (1985). High-molecular-weight components in lipopolysaccharides of *Salmonella typhimurium, Salmonella minnesota*, and *Escherichia coli*.. J Bacteriol.

[56] Grossman N, Schmetz MA, Foulds J, Klima EN, Jimenez-Lucho VE (1987). Lipopolysaccharide size and distribution determine serum resistance in *Salmonella montevideo*.. J Bacteriol.

[57] Tomas JM, Ciurana B, Benedi VJ, Juarez A (1988). Role of lipopolysaccharide and complement in susceptibility of *Escherichia coli* and *Salmonella typhimurium* to non-immune serum.. J Gen Microbiol.

[58] Bravo D, Silva C, Carter JA, Hoare A, Alvarez SA (2008). Growth-phase regulation of lipopolysaccharide O-antigen chain length influences serum resistance in serovars of Salmonella.. J Med Microbiol.

[59] Pescaretti Mde L, Lopez FE, Morero RD, Delgado MA (2011). The PmrA/PmrB regulatory system controls the expression of the *wzz/fepE* gene involved in the O-antigen synthesis of *Salmonella enterica* serovar Typhimurium.. Microbiology.

[60] Dawson JP, Weinger JS, Engelman DM (2002). Motifs of serine and threonine can drive association of transmembrane helices.. J Mol Biol.

[61] Senes A, Engel DE, DeGrado WF (2004). Folding of helical membrane proteins: the role of polar, GxxxG-like and proline motifs.. Curr Opin Struct Biol.

[62] Walters RF, DeGrado WF (2006). Helix-packing motifs in membrane proteins.. Proc Natl Acad Sci U S A.

[63] Alix E, Blanc-Potard AB (2008). Peptide-assisted degradation of the Salmonella MgtC virulence factor.. EMBO J.

[64] Nou X, Braaten B, Kaltenbach L, Low DA (1995). Differential binding of Lrp to two sets of pap DNA binding sites mediated by Pap I regulates Pap phase variation in *Escherichia coli*.. EMBO J.

[65] Holden NJ, Uhlin BE, Gally DL (2001). PapB paralogues and their effect on the phase variation of type 1 fimbriae in *Escherichia coli*.. Mol Microbiol.

[66] Baga M, Goransson M, Normark S, Uhlin BE (1985). Transcriptional activation of a *pap* pilus virulence operon from uropathogenic *Escherichia coli*.. EMBO J.

[67] White-Ziegler CA, Angus Hill ML, Braaten BA, van der Woude MW, Low DA (1998). Thermoregulation of *Escherichia coli pap* transcription: H-NS is a temperature-dependent DNA methylation blocking factor.. Mol Microbiol.

[68] Hung DL, Raivio TL, Jones CH, Silhavy TJ, Hultgren SJ (2001). Cpx signaling pathway monitors biogenesis and affects assembly and expression of P pili.. EMBO J.

[69] Lopez-Garrido J, Casadesus J (2010). Regulation of *Salmonella enterica* pathogenicity island 1 by DNA adenine methylation.. Genetics.

[70] Wallecha A, Munster V, Correnti J, Chan T, van der Woude M (2002). Dam- and OxyR-dependent phase variation of *agn43*: essential elements and evidence for a new role of DNA methylation.. J Bacteriol.

[71] Waldron DE, Owen P, Dorman CJ (2002). Competitive interaction of the OxyR DNA-binding protein and the Dam methylase at the antigen 43 gene regulatory region in *Escherichia coli*.. Mol Microbiol.

[72] Casjens SR (2008). Diversity among the tailed-bacteriophages that infect the Enterobacteriaceae.. Res Microbiol.

[73] Piao HH, Tam VT, Na HS, Kim HJ, Ryu PY (2010). Immunological responses induced by *asd* and *wzy/asd* mutant strains of *Salmonella enterica* serovar Typhimurium in BALB/c mice.. J Microbiol.

[74] Paixao TA, Roux CM, den Hartigh AB, Sankaran-Walters S, Dandekar S (2009). Establishment of systemic *Brucella melitensis* infection through the digestive tract requires urease, the type IV secretion system, and lipopolysaccharide O antigen.. Infect Immun.

[75] Holzer SU, Schlumberger MC, Jackel D, Hensel M (2009). Effect of the O-antigen length of lipopolysaccharide on the functions of Type III secretion systems in *Salmonella enterica*.. Infect Immun.

[76] Murray GL, Attridge SR, Morona R (2006). Altering the length of the lipopolysaccharide O antigen has an impact on the interaction of *Salmonella enterica* serovar Typhimurium with macrophages and complement.. J Bacteriol.

[77] Yoon H, Ansong C, Adkins JN, Heffron F (2011). Discovery of Salmonella virulence factors translocated via outer membrane vesicles to murine macrophages.. Infect Immun.

[78] Lahteenmaki K, Kyllonen P, Partanen L, Korhonen TK (2005). Antiprotease inactivation by *Salmonella enterica* released from infected macrophages.. Cell Microbiol.

[79] Hautefort I, Thompson A, Eriksson-Ygberg S, Parker ML, Lucchini S (2008). During infection of epithelial cells *Salmonella enterica* serovar Typhimurium undergoes a time-dependent transcriptional adaptation that results in simultaneous expression of three type 3 secretion systems.. Cell Microbiol.

[80] Morona R, Van Den Bosch L, Daniels C (2000). Evaluation of Wzz/MPA1/MPA2 proteins based on the presence of coiled-coil regions.. Microbiology 146 (Pt.

[81] Tocilj A, Munger C, Proteau A, Morona R, Purins L (2008). Bacterial polysaccharide co-polymerases share a common framework for control of polymer length.. Nat Struct Mol Biol.

[82] Helaine S, Thompson JA, Watson KG, Liu M, Boyle C (2010). Dynamics of intracellular bacterial replication at the single cell level.. Proc Natl Acad Sci U S A.

[83] Ackermann M, Stecher B, Freed NE, Songhet P, Hardt WD (2008). Self-destructive cooperation mediated by phenotypic noise.. Nature.

[84] Mastroeni P, Grant A, Restif O, Maskell D (2009). A dynamic view of the spread and intracellular distribution of *Salmonella enterica*.. Nat Rev Microbiol.

