# Supplementary material for: STM2209-STM2208 (opvAB): A Phase Variation Locus of Salmonella enterica Involved in Control of O-Antigen Chain Length
Source: PLoS One. 2012 May 11;7(5):e36863. doi: 10.1371/journal.pone.0036863 (PMC3350482; doi:10.1371/journal.pone.0036863)
Supplement: Table S1 — Oligonucleotides. (DOC) [file pone.0036863.s001.doc]

**Table S1**

| **Oligonucleotide name** | **Sequence (5’→3’)** |
| --- | --- |
| 2208mut1DIRnuevo | gaaccgtcacataaaacaaaaacCATCaattttatttatatgatag |
| 2208mut1INVnuevo | ctatcatataaataaaattGATGgtttttgttttatgtgacggttc |
| 2208mut23DIRnuevo | gaggaattttCATCgattttaattatttataacCATCgatatcatg |
| 2208mut23INVnuevo | catgatatcGATGgttataaataattaaaatcGATGaaaattcctc |
| 2208mut4DIRnuevo | ctatcattgatgtatttaccCATCgatataaccagtgtgaatgtattg |
| 2208mut4INVnuevo | caatacattcacactggttatatcGATGggtaaatacatcaatgatag |
| Clo2208-3 | AGTCGAGCTCCCATCATCGATATACCATGC |
| Clo2208-5 | CGACTCTAGACTGATCATGATGACGTCCAC |
| ClooxyR-3 | TTTTtctagaTAACGCCTTGTCGAAATGGC |
| ClooxyR-5 | TTTTgagctcGAATATCTGGTGGCGTTAGC |
| delGATC-PS1 | ccccgttggtgcctgaacgtgtaccgaatgaaccgtcacaGTGTAGGCTGGAGCTGCTTC |
| delGATC-PS2 | agaattcgagtattttaaaggaaaataacaatacattcacCATATGAATATCCTCCTTAG |
| deloxyR199-PS1 | ccgatctggcgggcgagaaattgctgatgctggaagatggGTGTAGGCTGGAGCTGCTTC |
| deloxyR199-PS2 | tccgctcccgcttcaaaacagaaccccatcgcctgatcgcCATATGAATATCCTCCTTAG |
| F2208-3 | ttcgacacatttcagcgcagagtttatctctgcgcaatgtCATATGAATATCCTCCTTAG |
| F2208-5 | agaatatcgtattgagaaaaagacaatgaatgaccgcgcaGACTACAAAGACCATGACGG |
| F2209-3 | aacgtcgactaaatcaatttcactattttctccccgcattCATATGAATATCCTCCTTAG |
| F2209-5 | ttcagtattcgggttgactattagcgttttaaaagggatgGACTACAAAGACCATGACGG |
| neo-lacZ | GCTGCAAGGCGATTAAGTTG |
| oxyR3-PS2 | gtacgacgcggctccggcttaatgcatggcagataaaccaCATATGAATATCCTCCTTAG |
| oxyR5-PS1 | actgcgtgaggtcaaggtgctcaaggagatggcaagccaaGTGTAGGCTGGAGCTGCTTC |
| oxyRC199SDIR | ggaagatggccactCtctgcgcgatcagg |
| oxyRC199SINV | cctgatcgcgcagaGagtggccatcttcc |
| oxyR-E1 | ggttaaacgagaaaccgctc |
| oxyR-E2 | cacctttaactacccaacc |
| PE2208 | CGACGGATCCaaggaaacgtcgactaaatc |
| PE2209 | CGACGGATCCctgcgaacgtatatttcttc |
| PE5 | ATTAGGATCCagccttgtcttcggaatgtc |
| pKT25-seq3 | CTGCAAGGCGATTAAGTTGG |
| pKT25-seq5 | TTATGCCGCATCTGTCCAAC |
| pKT25-STM2208-PstI-5 | AActgcagggATGCGGGGAGAAAATAGTG |
| pKT25-STM2209-BamHI-3 | CGggatccTCACATCCCTTTTAAAACG |
| pKT25-STM2209-PstI-5 | AActgcagggATGAAGAAATATACGTTCG |
| pUT18C-STM2208-BamHI-3 | CGggatccTCATGCGCGGTCATTCATTG |
| pUT18C-STM2208-PstI-5 | AActgcaggATGCGGGGAGAAAATAGTG |
| pUT18C-STM2209-PstI-5 | AActgcaggATGAAGAAATATACGTTCG |
| RT2208-3 | agctttgcatatgtttccgtttg |
| RT2208-5 | aatggcggcatggtatatcg |
| RT2209-3 | gctaatagtcaacccgaatac |
| RT2209-5 | gaagaaatatacgttcgcagc |
| STM2208-E1 | aatatacgttcgcagccagg |
| STM2208-E2 | ttcgacacatttcagcgcag |
| STM2208-PS1 | gcgcggtcattcattgtctttttctcaatacgatattctgGTGTAGGCTGGAGCTGCTTC |
| STM2208-PS4 | gggagaaaatagtgaaattgatttagtcgacgtttccttaATTCCGGGGATCCGTCGACC |
| STM2209-E1 | ttaccgatcgatataaccag |
| STM2209-E2 | ttgtatcatgctgcacgctc |
| STM2209-PS1 | tttcactattttctccccgcatttcacatcccttttaaaaGTGTAGGCTGGAGCTGCTTC |
| STM2209-PS4bis | gttgctttttgttatttcagtattcgggttgactattagcATTCCGGGGATCCGTCGACC |
